# Sulfotransferase 4A1 Coding Sequence and Protein Structure Are Highly Conserved in Vertebrates

**DOI:** 10.3390/genes15070914

**Published:** 2024-07-13

**Authors:** Robert C. A. M. van Waardenburg, Charles N. Falany

**Affiliations:** Department of Pharmacology and Toxicology, University of Alabama at Birmingham, Birmingham, AL 35294, USA; cfalany@uab.edu

**Keywords:** gene structure, polymorphism, protein structure, tissue/cell distribution

## Abstract

Cytosolic sulfotransferases (SULTs) are Phase 2 drug-metabolizing enzymes that catalyze the conjugation of sulfonate to endogenous and xenobiotic compounds, increasing their hydrophilicity and excretion from cells. To date, 13 human SULTs have been identified and classified into five families. SULT4A1 mRNA encodes two variants: (1) the wild type, encoding a 284 amino acid, ~33 kDa protein, and (2) an alternative spliced variant resulting from a 126 bp insert between exon 6 and 7, which introduces a premature stop codon that enhances nonsense-mediated decay. SULT4A1 is classified as an SULT based on sequence and structural similarities, including PAPS-domains, active-site His, and the dimerization domain; however, the catalytic pocket lid ‘Loop 3’ size is not conserved. SULT4A1 is uniquely expressed in the brain and localized in the cytosol and mitochondria. *SULT4A1* is highly conserved, with rare intronic polymorphisms that have no outward manifestations. However, the *SULT4A1* haplotype is correlated with Phelan–McDermid syndrome and schizophrenia. SULT4A1 knockdown revealed potential SULT4A1 functions in photoreceptor signaling and knockout mice display hampered neuronal development and behavior. Mouse and yeast models revealed that SULT4A1 protects the mitochondria from endogenously and exogenously induced oxidative stress and stimulates cell division, promoting dendritic spines’ formation and synaptic transmission. To date, no physiological enzymatic activity has been associated with SULT4A1.

## 1. Introduction

Sulfation is a major reaction in human Phase 2 drug/xenobiotic and endogenous compound metabolism [1]. The cytosolic sulfotransferase (SULT) multigene family is responsible for drug/xenobiotic sulfation (Table 1). A separate SULT multigene family of membrane-associated Golgi SULTs are responsible for the glycosaminoglycan/glycoprotein sulfation that occurs in the Golgi apparatus [1,2,3,4,5]. Both families of enzymes utilize 3′-phosphoadenosine 5′-phosphosulfate (PAPS) as the obligate sulfonate donor.

**Figure 1:**
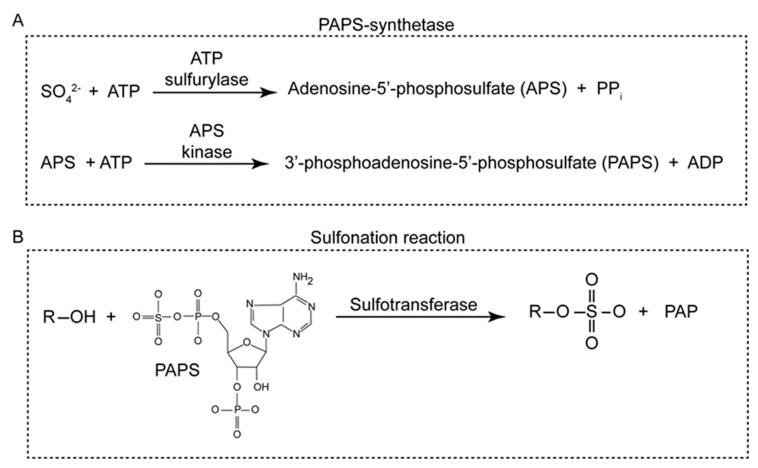
Schematic reaction mechanism for general cytosolic sulfotransferase action. (**A**) 3′-Phosphoadenosine 5′-phosphosulfate (PAPS) synthesis is a two-step reaction that in vertebrates is catalyzed by one enzyme, PAPS synthetase (PAPSS). Human cells have two isoforms: PAPSS1 and PAPSS2. Generation of PAPS involves two ATP and one sulfate molecule. (**B**) General mechanism of cytosolic SULT-catalyzed sulfonation of a molecule (R-SO_4_) involves transfer of sulfonate (donated by co-factor PAPS) to a hydroxyl-group of the substrate molecule (R-OH), e.g., phenol, cholesterol, or steroid.

PAPS is synthesized in every organism using one sulfate (SO_4_^−2^) and two ATP molecules in a two-step process either facilitated by one or two enzymes (Figure 1A). In fungi, bacteria, and plants, ATP sulfurylase and APS kinase activities reside in separate polypeptide chains. In metazoans, these two peptides have been combined into a single bifunctional protein termed PAPS-synthetase. In the ATP-sulfurylase reaction, the reversible synthesis of APS is kinetically unfavorable, and the reaction is pulled by the removal of the PPi to stimulate PAPS synthesis [6,7]. The “sulfation” reaction primarily involves the transfer of a sulfonate (SO_3_^2−^) group from PAPS to a hydroxyl group on the substrate to form a sulfate, with 3′,5′-diphosphoadenosine (PAP) as a by-product (Figure 1B). However, the sulfonation reaction has historically been termed “sulfation” following the initial identification of phenol sulfate formation in human patients [1].

## 2. SULT4A1 Discovery

**Figure 2:**
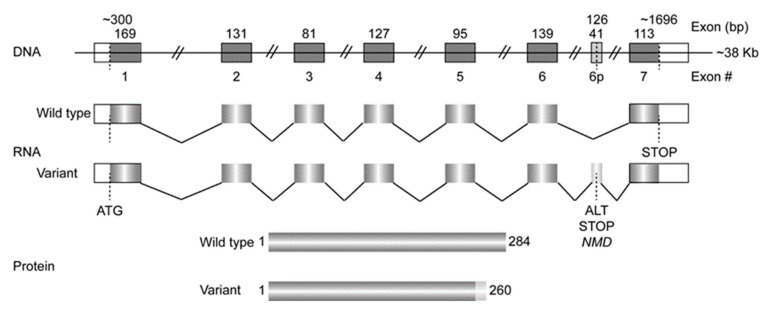
SULT4A1 gene and mRNA structure. Gene structure of human SULT4A1 exhibits 7 exons and 6 introns, with intron 6 housing an alternative exon 6p that is inserted by alternative splicing and results in a premature stop codon that primes the SULT4A1 variant 1 mRNA for nonsense-mediated mRNA decay (NMD). This alternative splicing mechanism regulates expression of the wild-type SULT4A1 protein of 284 amino acids in all tissues. The variant 1 mRNA, if translated, would express a 260 amino acid protein that is identical to the wild-type protein until Arg248, in loss of the dimerization domain in its alternative C-terminal domain.

Several SULT isoforms are included in the cytosolic SULT family, although definitive proof of their SULT catalytic activity has not been described [8,9,10]. SULT4A1 has been included in the superfamily based on its sequence and subsequent structural similarity to other cytosolic SULTs, although PAPS-dependent catalytic activity has not yet been described [3,8]. A partial SULT4A1 mRNA was initially isolated from a human pancreatic β-cell library based on several general SULT conserved sequences [11]. Attempts to isolate a full-length translatable message from the library were unsuccessful [12]. Subsequently, the Northern blot probing of a human multi-tissue RNA blot detected an intense specific signal in human brain RNA [8]. The brain message translated a 284 amino acid (aa) protein sequence that possessed several conserved SULT sequences, including PAPS binding domains, an active site His residue, and the dimerization domain [8]. This similarity to other SULTs led to the inclusion of SULT4A1 in the SULT superfamily [8,9,13]. In the original report, an alternative cDNA sequence was also isolated from a human liver cDNA library [8]. The alternative liver cDNA was identical in sequence to the brain cDNA but had a 126 bp insertion between exon 6 and 7 (Figure 2) [8]. The 126 bp sequence was inserted via the alternative splicing of intron 6 with the alternative exon termed ‘6p’, generating a premature stop codon that would encode a 260-aa protein [8,14].

## 3. SULT4A1 Genetics

**Figure 3:**
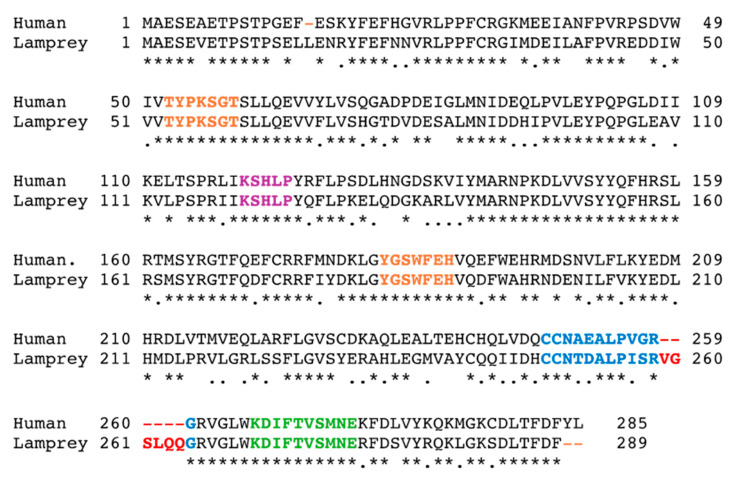
Alignment of the SULT4A1 from lamprey with the human SULT4A1 amino acid sequence. Lamprey, an early primitive vertebrate, contains the oldest known SULT4A1 protein with an amino acid sequence that is highly conserved through its evolution to human SULT4A1 (69% identical). PAPS binding pocket residues (orange), active site His domain (Cyan), and the dimerization domain (green). The residues of the Loop 3 catalytic pocket lid (Blue) with the additional residues (Red) that are in Loop 3 of Lamprey SULT4A1 but were seemingly lost in the subsequent evolutionary step to sharks. *: identical amino acids, .: similar amino acid.

The human SULT family comprises 13 members. Eleven *SULT*-genes are in four chromosomal clusters (isoforms of SULTs 1As, 1B/1E, 1Cs, and 2A/2B subfamily), and the two most recently discovered SULT genes occur at single gene loci (4A1 and 6B1) (Table 1). We will focus on *SULT4A1* polymorphisms/haplotypes and their potential relation to human disease, while the evolution and pharmacogenomics of the ‘older’ SULT isoforms have been extensively reviewed before [9,10,15,16,17]. Phylograms illustrate that *SULT4A1* might have originated from the beginning of *SULT* genes [15,17]. SULT4A1 homologues, with the human SULT4A1 aa sequence as a query (https://blast.ncbi.nlm.nih.gov, access on 2 May 2024), were found in the genome sequences starting with lampreys (Chordata, Agnatha, Hyperoartia, Petromyzontiformes, and Petromyzontidae), the earliest (primitive) vertebrate organism that has a defined skull (craniate) and vertebral column (Figure 3).

We did not find any homologous SULT4A1 sequences in annotated hagfish (Chordata, Agnatha, Myxini, Myxiniformes, and Myxinidae) genomes during our blastp search using human SULT4A1 aa as a query. Hagfish are close relatives to lampreys and have a primitive brain and neuronal cord with an underdeveloped skull. Alignment of the human and lamprey SULT4A1 protein sequences revealed a highly conserved amino acid sequence with 69% aa identity conservation (Figure 3). Moreover, the PAPS binding region, the active site His-patch, and the dimerization domain were fully conserved between human and lamprey SULT4A1. Intriguingly, the SULT-active pocket lid, Loop 3 (discussed in more detail below), of lamprey SULT4A1 contains an additional six residues compared to all other SULT4A1 homologs (Figure 3). This suggests that, in the evolutionary step from lampreys (Petromyzontida) to sharks (Chondrichthyes), these six residues were lost. Following this evolutionary change, the SULT4A1 protein sequence underwent only minor changes during evolution from the currently earliest SULT4A1 sequence in lamprey to humans. This is reflected in the high percentage of identical amino acids, from 69% for lamprey to 87% for sharks and 97.9% for mice compared to humans (Table 2). 

**Figure 4:**
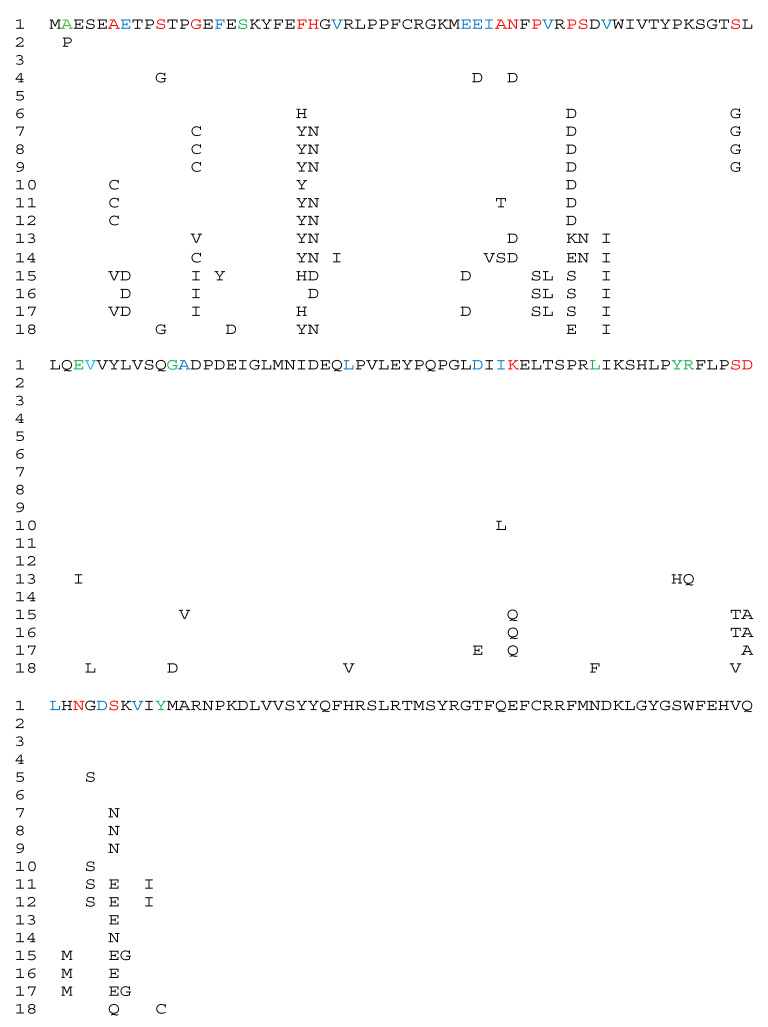

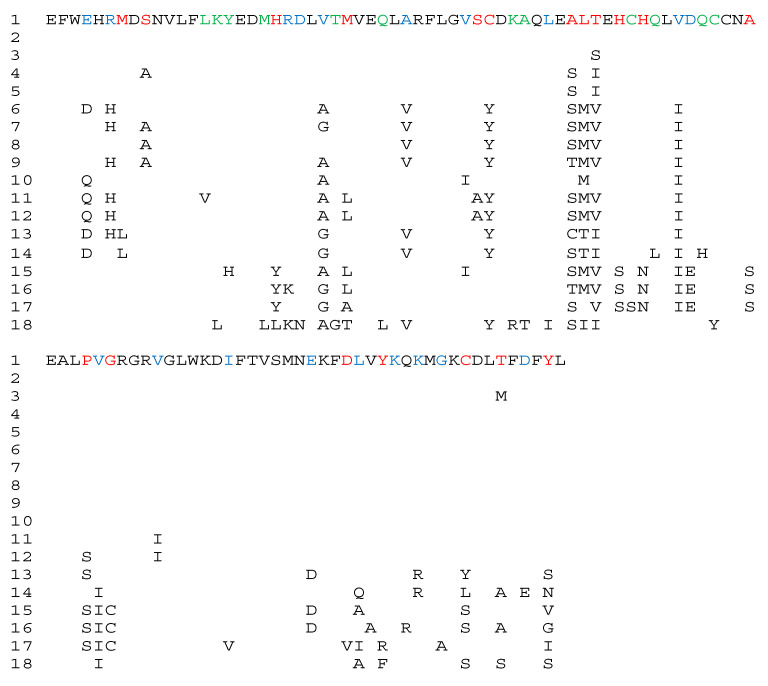
Alignment of vertebrate SULT4A1 amino acid sequences. (1) Human, (2) Pongo, (3) Red Fox, (4) Mouse, (5) Sperm whale, (6) Short-tailed opossum, (7) Great Tit, (8) Zebrafinch, (9) European Starling, (10) Anole lizard, (11) Brown Pit Viper, (12) Eastern Brown Snake, (13) High Himalayan Frog, (14) Xenopus, (15) Catfish, (16) Northern Pike, (17) Zebrafish, and (18) Whale shark. Blue is conserved, Red is multiple changes, and Green is a single change in one of the 18 species. SULT4A1 aa sequence identity conservation shown in Table 2.

The SULT4A1 vertebrate aa sequence alignment is shown in Figure 4. Moreover, in surveying the evolution from SULT4A1 in lamprey to humans, many of the differences in the amino acid sequence are substitutions with a ‘similar’ amino acid. Interestingly, many of these substitutions appear in multiple organisms, suggesting an adaptive change to a potential substrate(s).

This suggests that the function of SULT4A1 is highly conserved and sensitive to sequence changes. The latter is supported by the observation that, for human *SULT4A1*, only 17 single-nucleotide polymorphisms (SNPs) are detected (Table 3), which is a remarkably low number for a 38Kb gene locus. Especially as it was reported that, for human chromosome 22, one SNP was detected every 1.07 Kb, with 18% of the potential variants being insertions or deletions [18]. Moreover, all *SULT4A1* SNPs are positioned in the non-coding intron regions of the gene and do not display directly related clinical manifestations. The only clinical manifestations that might be correlated with the *SULT4A1* polymorphism/haplotype are the psychopathological disorders Phelan–McDermid syndrome and schizophrenia [17,19,20,21,22,23,24]. Multiple studies have suggested that the *SULT4A1-1* haplotype (bolded SNPs in Table 3) might be correlated with schizophrenia susceptibility, yet *SULT4A1-1*(+) haplotype patients display an improved response to treatment and reduced hospitalization rates [20,22,25]. However, a large retrospective analysis showed that the SULT4A1 haplotype is not significantly correlated with schizophrenia susceptibility and response to treatment [26]. Phelan–McDermid syndrome is correlated with the deletion of *SHANK3* within the chromosome 22q13.3 band and an additional six gene deletions/variants, including *SULT4A1* [24]. A recent Phelan–McDermid syndrome mechanism of pathology review demonstrated that the seven involved genes influence some combination of stress, inflammation, injury response, and mitochondria function [24]. The loss of SULT4A1 known physiological functions, including the protection of mitochondria from oxidative stress [27,28], interaction with PIN1 [29], the regulation of dendritic spine formation, and synaptic transmission by promoting PSD-95/NMDAR complex formation [30], affects SHANK3 protein levels and post-synaptic density, which are part of Phelan–McDermid syndrome etiology [24,30]. In addition, SULT4A1 is a significant pathological candidate in cases of SHANK3-independent Phelan–McDermid syndrome [31]. Therefore, it is probable that SULT4A1-associated function affects the Phelan–McDermid syndrome phenotype from its developmental stages to maturity. Thus, whatever the function and activity of SULT4A1, it is highly conserved and significant, and does not appear to tolerate any minor changes in the protein. This became obvious from the neuronal-related symptoms the two different strains of Sult4A1 knockout mice displayed, resulting in death within 25 days (discussed below) [28,32]. In perspective, since no direct SULT4A1 activity has been reported, we and others can only speculate based on reported associations about the underlying mechanism and contribution of SULT4A1 to these neuronal pathological syndromes. Moreover, most patients that present these syndromes have additional genetic alterations/deletions, such as in case of Phelan–McDermid syndrome at the *SULT4A1* region 22q13, which also include *ATXN10, BRD1, CELSR1, MLC1, MAPK8IP2, PHF21B, SHANK3*, and *TCF20*. A combination of these gene defects/deletions and other genes outside the 22q13 region is likely to contribute to Phelan–McDermid syndrome development and schizophrenia susceptibility. Mitz et al., in their 2024 review [23], describe, in depth, the loss of a combination of the known overlapping functions of these proteins and the reported contribution of SULT4A1′s potential function in spine formation, synaptic transmission, and mitochondrial health. They reason that impaired responses to oxidative and/or inflammation stress and injuries to mitochondrial function contribute to the neurological pathology of these syndromes [23]. On the other hand, the conflicting observations that SULT4A1 haplotypes affect schizophrenia treatment [26] might be related to the currently assumed sulfonation activity of SULT4A1 [8,27] and of therapeutic agents and their additional modification by, e.g., CYP P450. 

## 4. SULT4A1 Expression and Tissue Distribution

Heterologous expression of the human and mouse *SULT4A1* cDNAs in *Escherichia coli* resulted in a ~33 kDa protein which was immunologically similar to a protein in mouse and human brain cytosol [33]. This immunohistochemical study detected strong SULT4A1 protein signals in distinct areas: the brainstem, cerebellum, cerebral cortex, and pituitary [33]. Although SULT4A1 messages can be detected in many other human and rat tissues by Northern blotting or RT-PCR, the mRNA appears to be only appropriately spliced and translated in neurons [34]. Other tissues seem to express the alternatively spliced SULT4A1 mRNA containing an additional exon 6p, which introduce a premature stop codon (Figure 2). Moreover, the introduced premature stop codon generates an mRNA that is primed for nonsense-mediated mRNA decay [14,35]. If this variant of SULT4A1 mRNA was able to be translated, it would encode a 260-aa protein that is identical to the wild-type SULT4A1 protein up to Arg248. This variant of SULT4A1 would not contain the conserved SULT dimerization domain and the SULT4A1 C-terminal tail (Figure 2). For SULT4A1 in general, inappropriate splicing leads to the rapid degradation of the partial SULT4A1 transcripts with remaining mRNA levels still detectable by RT-PCR. The generation of short in-frame deletions in the SULT4A gene results in the apparent rapid degradation of the expressed protein, as analyzed in mice and zebrafish by immunoblotting [32,36]. Sidharthan et al. demonstrated that SULT4A1 messages are alternatively spliced in the SH-SY5Y and SK-N-MC neuroblastoma cell lines; however, upon retinoic acid differentiation, the SULT4A1 mRNA was properly spliced and the SULT4A1 protein was detected [37]. This observation suggests that SULT4A1 expression is dependent on specific splicing factors that are expressed upon the differentiation of these cells to a neuronal phenotype. In SH-SY5Y cells, it was shown that the MBNL and CELF RNA-processing factors are capable of the appropriate splicing of SULT4A1 messages [38]. However, these splicing factors are widely expressed in human tissues, suggesting that the specificity for the proper splicing of the SULT4A1 message is still unknown [38]. Moreover, Colombrita et al. showed that, in another neuroblastoma cell line, SK-N-BE, the RNA-binding protein FUS is responsible for the correct SULT4A1 splicing and not TDP-43 [39]. Furthermore, these authors used an array of RT-PCR primers distributed over the SULT4A1 mRNA and demonstrated that RT-PCR primers amplifying the exon 6–exon 7 fusion boundary will discriminate between wild-type and variant SULT4A1 mRNA. This exon 6–7 primer set should be the standard in all mRNA expression screens to obtain a correct overview of wild-type and variant SULT4A1 mRNA expression and potentially the SULT4A1 protein to appropriately determine SULT4A1 mRNA tissue distribution. 

## 5. SULT4A1 Protein Folding and Structure

**Figure 5:**
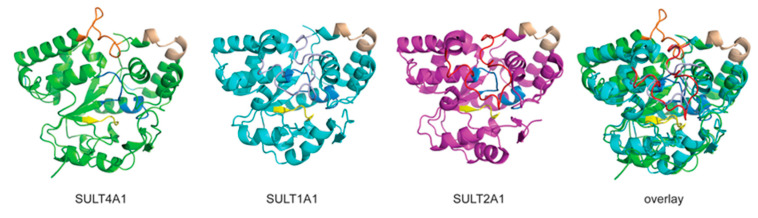
Structural comparison of Loop 3 between SULT4A1 and the conserved SULTs 1A1 and 2A1. Shown is a cartoon representation of the resolved crystal protein structures of SULT1A1 (PDB 4GRA), SULT2A1 (PDB 3F3Y), and SULT4A1 (PDB 1ZD1) with the prediction model of the 4A1 N-terminal domain attached (unpublished data Tibbs and Falany). The overlay of all three SULTs (not shown is the protein cartoon of SULT2A1 for clarity) to highlight the difference between the highly conserved SULT Loop 3 and unique SULT4A1 loop 3, or catalytic pocket lid. Loop 3 for 4A1 (Orange), 1A1 (Light blue) and 2A1 (Red). Conserved domains are highlighted in blue (PAPS binding pocket), yellow (active site His domain), and wheat (dimerization domain). All structure cartoons were generated using PyMol 2.5.2 (Molecular Graphics System, Schrödinger, LLC, New York, NY, USA).

SULT4A1 is included in the cytosolic SULT gene family based on its sequence and structural similarity to the other SULT family members [3,8]. Figure 5 shows the structural alignment of SULT4A1 (PDB 1ZD1) and the major human phenol and steroid SULTs, SULT1A1 (PDB 4GRA) and SULT2A1 (PDB 3F3Y), with conserved domains highlighted [3]. All SULTs, including SULT4A1, possess the “TYPKSGT” and “YGSWXEH” PAPS-binding domains (Figure 5 blue), an “(K)SHLP” active site His domain (Figure 5, yellow), and the conserved “KXXXTVXXXE” dimerization domain (Figure 5, wheat). However, one obvious distinguishing characteristic is the gap in its sequence, which is present in all other cytosolic SULT isoforms and known as ‘Loop 3” [3]. Loop 3 forms the outer surface of the PAPS- and substrate-binding pockets and functions as a lid over the catalytic pocket that can open and close [40,41]. These differences are highlighted in Figure 5’s ‘overlay’ of Loop 3 for 4A1 in orange, 1A1 in light blue, and 2A1 in red in the context of the cartoon of SULT4A1 and 1A1 (2A1 not shown for clarity). Loop 3, in the absence of PAPS/PAP binding, is highly flexible and does not resolve in the resolved structures [3]. Only SULT2A1 structures were resolved with a partial Loop 3 in the absence of bound PAPS/PAP and the presence of DHEA [42]. In the presence of PAPS/PAP, Loop 3 is restructured and stabilized, allowing for a resolution of this region [3]. To date, a single resolved structure for SULT4A1 has been reported (PDB 1ZD1) [3]. The expressed protein used to generate the structure did not bind PAPS or PAP, and PAPS was not identified in the active site of the resolved structure. As shown in Figure 5, the SULT4A1 structure is very similar to that of SULT1A1 and SULT2A1.

**Figure 6:**
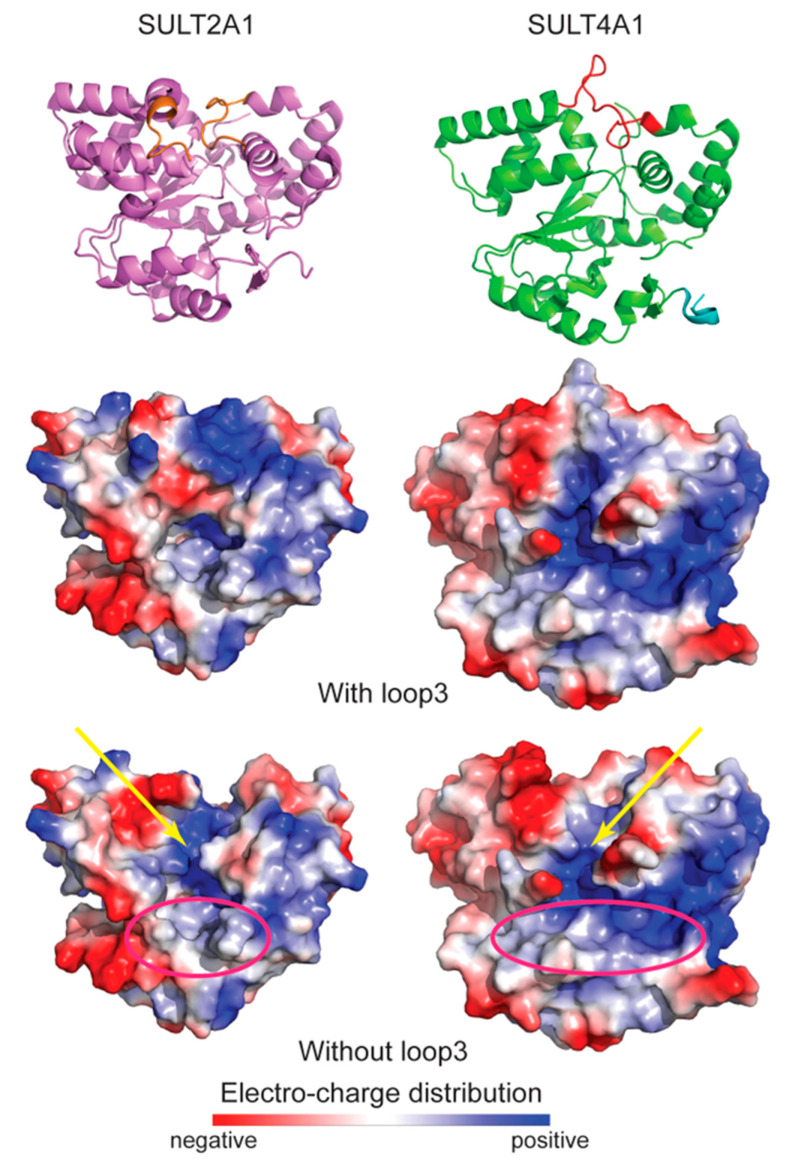
Surface electro-charge distribution comparison of SULTs 2A1 and 4A1. Resolved crystal protein structures of SULT2A1 (PDB 2QP4) (without PAP(S) but with DHEA) and SULT4A1 (PDB 1ZD1) displayed as cartoon (with Loop 3 in orange for 2A1 and in red for 4A1) and electro-charge surface with or without (bottom) Loop 3 [3,42]. All structure cartoons and electro-charge displays were generated using PyMol 2.5.2 (Molecular Graphics System, Schrödinger, LLC). Yellow arrow points to the electro-charge distribution of the PAPS-binding site in ‘opened’ catalytic pockets. Pink circle shows ‘substrate-binding site’.

The major differences between SULT4A1 and other SULTs are that the SULT4A1 shortened Loop 3 does not cover the active site and active site His residue, the substrate binding pocket, and most of the PAPS binding pocket appears exposed to the solvent. Loop 3 changes the electro-charge distribution of the SULTs’ surfaces. For example, Loop 3 provides SULT2A1 (PDB 2QP4) with a more positively charged surface and SULT4A1 with a more neutrally charged surface (Figure 6) [42]. If we remove Loop 3 and ‘open’ the catalytic pockets, the electro-charge distribution of the PAPS-binding site (yellow arrow, Figure 6) is similar between SULT4A1 and SULT2A1, while the ‘substrate-binding site’ (pink circle, Figure 6) shows a similar charge distribution but differences in geometry that are narrower for SULT2A1 and more open and less contained for SULT4A1. The latter might point to differences in substrate sizes between SULT4A1 and the other cytosolic SULTs. However, the SULT4A1 structure (PDB 1ZD1) does not show its N-terminal domain (NTD), suggesting that it is very dynamic, and its modeled structure is shown in Figure 5. The molecular modeling of SULT4A1′s flexible NTD suggests that it might be able to cover the substrate binding pocket, at least partly, suggesting that the NTD can regulate substrate binding. Moreover, the SULT4A1 NTD contains two Thr (T8 and T11) residues, with Thr11 being phosphorylated by Erk1 kinase and dephosphorylated by serine/threonine protein phosphatase 2A (PP2A) [43]. Thr11 phosphorylation stimulates the interaction with peptidyl-prolyl cis-trans isomerase PIN1, resulting in a decrease in SULT4A1 protein stability [29]. This observation suggests that SULT4A1 could act as a scavenger for PIN1 in, for example, neurons to prevent PIN1 from interacting with the NMDA receptor, as such facilitating NMDA:PSD-95 interactions and reducing SHANK3 ubiquitination [23].

## 6. SULT4A1 Tissue and Cellular Distribution, Physiological Role

SULT4A1 is predominantly expressed throughout the brain [33], yet we still do not know its physiological function, enzyme activity, or substrate(s). Moreover, SULT4A1 did not bind or retain PAPS/PAP in vitro [3]. This contrasts with other cytosolic SULTs that are expressed in multiple tissues [44]. Liyou et al. reported the first detailed immunohistochemical expression map of human and rat SULT4A1 protein levels [33,34]. They observed that, in humans and rats, the SULT4A1 protein is exclusively expressed in the brain. The strongest immunostaining was detected in the following brain regions: the cerebellum, cerebral cortex, brain stem, and pituitary, suggesting a function for SULT4A1 in the central nervous system [33]. Crittenden et al. reported the first linked cellular process, phototransduction, to SULT4A1 using zebrafish [45]. Deep sequencing following a morpholino-mediated knockdown of SULT4A1 in zebrafish larvae (72-hpf) resulted in the upregulation of 14 photoreceptor-related genes [45]. In addition, SULT4A1 knockdown affected the gene expression related to LXR/RXR activation, circadian rhythm signaling, and neuronal CREB signaling in zebrafish [45]. These observations are potentially correlated with the human SULT4A1 haplotypes linked to psychopathologic diseases such as schizophrenia and Phelan–McDermid syndrome [24,25,46]. A transient 72 h SULT4A1 knockdown had no effect on development and did not show any gross phenotypic differences with control wild-type larvae. However, this study did not follow the fish to the adult stage, where potential behavioral changes might emerge. Subsequently, Crittenden et al. generated a *SULT4A1* knockout zebrafish with an eight-nucleotide deletion in *SULT4A1* exon 2, resulting in a frameshift and premature stop codon [36]. The *SULT4A1* knockout zebrafish showed excessive sedentary behavior, with increased inactivity bouts both in length and frequency and a general decrease in ‘daytime’ activity compared to wild-type zebrafish [36]. It is still unknown if these *SULT4A1*^Δ8/Δ8^ phenotypes are related to their previously observed dysregulation of cone genes in transient SULT4A1 knockdown larvae codon [36,45]. Although zebrafish seems to be a good model for the study of SULT4A1, the water barrier and size do hinder neuronal studies. Subsequently, the Falany lab developed two different knockout mice, one exhibiting a 28 bp deletion in exon 1, and the other mouse possessing a 12 bp deletion immediately preceding the potential active site His111, with both deletions resulting in strong *SULT4A1* knockout phenotypes [32]. Pups of both strains with a homozygous SULT4A1 deletion demonstrated severe and progressive neurological symptoms, including absence seizures, tremors, abnormal gait, ataxia, and a decreased weight gain, leading to postnatal death within 21–25 days compared to wild-type littermates [32]. These early deaths were due to an inability to consume solid rodent chow. Additionally, SULT4A1 demonstrates a different cellular distribution from other cytosolic SULTs, with SULT4A1 detected in the cytosolic, mitochondrial, and microsomal fractions, but not in the nuclear fraction [28,32]. Mitochondrial association was detected via the immunohistochemical staining of primary mice neurons and the immunoblotting of a subcellular fractionation of differentiated SH-SY5Y cell extracts [28]. Furthermore, SULT4A1 shRNA-mediated depletion in mouse cortical neurons induced ROS accumulation, as determined by life-cell imaging with CellRox Deep Red [28]. This suggests that SULT4A1 may regulate mitochondrial homeostasis, since mitochondria are major generators of ROS levels, specifically in neuronal cells [47]. Subsequently, it has been shown that the homologous expression of SULT4A1 in undifferentiated SH-SY5Y neuroblastoma cells protects against mitochondrial hydrogen-peroxide-induced oxidative stress and cell death [28]. Additionally, Seahorse flux analysis demonstrated that, in cultured mouse cortical neurons, SULT4A1 increased the maximal oxygen consumption rate and spare respiratory capacity and protected against oxidative-stress-induced ATP turnover [28]. Moreover, SULT4A1 expression preserved the mitochondrial membrane potential following hydrogen peroxide treatment, yet did not affect the observed proton leakage [28]. Overall, these studies are the first to show that SULT4A1 plays a role in mitochondrial function and viability and protection toward oxidative stress, via a to-be-elucidated mechanism that is critical to neurons, as they have high bioenergetic demands. To study the function and activity of SULT4A1 on a molecular level, we validated and utilized the single-cell model organism *Saccharomyces cerevisiae* or budding yeast [27]. Budding yeast is a uniquely situated model organism to study SULTs, as it naturally generates PAPS. The PAPS in yeast is not the sulfonate donor for SULT activity, but functions as the sulfate donor for Met and Cys biosynthesis [48]. Moreover, yeast does not demonstrate any SULT activity or show any homologous SULT gene sequences. The heterologous expression of SULT4A1 is well tolerated by yeast, produces a stable protein without any negative effects on growth, and demonstrates a similar cellular distribution to that in human brain and mouse cortical neurons [27]. Additionally, the expression of SULT4A1 protects yeast against endogenous and exogenous oxidative stress in colony formation assays [27]. Furthermore, SULT4A1 in yeast is located in the cytosol and binds to the mitochondrial outer membrane at the cytosolic side, as determined via the immunoblotting of the subcellular fractionation fraction and isolated mitochondria treated with or without trypsin [27]. SULT4A1 expression in yeast stimulates colony formation under fermentative (aerobic glycolysis or Warburg effect) and respiratory growth conditions, which could be related to the reported SULT4A1-stimulated formation of neuronal branching and dendritic spines [27,30]. An immunohistochemical analysis of shRNA-mediated SULT4A1 knockdown in rat cortical neurons and brain slices of in utero electroporated mice resulted in a decrease in spine numbers, but did not affect neuron morphology [30]. This growth stimulation is possibly related to SULT4A1-mediated improved mitochondria activity via a currently unknown mechanism. In yeast, sulfate is assimilated into the universal SULT sulfonate donor PAPS, which yeast uses as a sulfur donor to produce Met and Cys. Importantly, all the observed yeast functional phenotypes are sulfate dependent, suggesting, for the first time, that SULT4A1 possesses SULT activity. Speculatively, these observations could indicate that SULT4A1 protects mitochondria that stimulate neuronal cell propagation during aerobic glycolysis, which peaks during early childhood and becomes more restricted to specific areas during adulthood, coinciding with the observed increase in SULT4A1 expression during development [27,49,50,51]. 

## 7. In Summary

Neuron-specific SULT4A1 is still an enigma approaching its 25-year discovery anniversary. However, this orphan SULT is slowly revealing its function and activity. While no physiological substrate or sulfonation activity has been detected, evolutionarily, the vertebrate-specific SULT amino acid sequence is remarkably conserved from the earliest ortholog of lamprey to the current human isoform. SULT4A1 expression in human tissue is regulated via the expression of RNA splicing proteins that regulate the alternative splicing of its messenger RNA by the introduction of an additional exon, resulting in a premature stop codon that stimulates nonsense-mediated mRNA decay. SULT4A1 haplotypes and other genomic variations have been correlated with some psychopathological disorders. Moreover, heterologous expression, knockdown, and knockout studies in cell lines, zebrafish, rat, and mouse models have revealed that SULT4A1 plays a significant role in neuronal function. Cell line models, mouse and rat cortical neurons, and the single-cell model *S. cerevisiae* have revealed that SULT4A1 is able to stimulate cell growth, including dendritic spine formation, and stimulate mitochondrial function and protection against endogenous and exogenous oxidative stress. Although we are slowly uncovering the functions in animal models and human cell line models, we are encouraged that our yeast-based studies will provide insight into the enzymatic activity and substrate selection of this intriguing SULT. In the near future, there is a critical need to elucidate the molecular activity of SULT4A1 to begin to understand the underlying mechanisms of the many SULT4A1-associated functional phenotypes. SULT4A1 is primed to act as a sulfotransferase, as suggested by the sulfate-dependent SULT4A1-related phenotypes in yeast, its protein sequence, and structure conservation with known SULTs. Moreover, there is potential that SULT4A1 exhibits a non-SULT function, such as, e.g., a scaffold protein, as suggested by SULT4A1 binding to the mitochondria out member, while SULYT4A1 does not show membrane anchor characteristics, suggesting a potential SULTY4A1–protein(s) interaction(s). Additionally, SULT4A1 interacts with proteins such as PIN1, acting as a scavenger that prevents PIN1 from affecting PSD-95/NMDAR complex function [23,30]. 

## Figures and Tables

**Table 1:** Chromosomal location of human SULT isoforms ^a^.

Gene	Chromosome	Location	Size ^b^	Strand	Protein (Da)
SULT1C2	2q12.3	108,288,639–108,309,915	21,277	+	34,880
SULT1C3	2q12.3	108,239,968–108,265,351	25,384	+	35,889
SULT1C4	2q12.3	108,377,911–108,388,989	11,079	+	35,520
SULT6B1	2q22.2	37,167,820–37,196,598	28,779	-	34,919
SULT1B1	4q13.3	69,721,167–69,787,961	66,795	-	34,899
SULT1E1	4q13.3	69,821,122–69,860,145	39,024	-	35,126
SULT1A1	16p11.2	28,605,196–28,623,375	18,180	-	34,165
SULY1A2	16p11.2	28,591,943–28,597,050	5108	-	34,310
SULT1A3	16p11.2	30,199,228–30,204,310	5083	+	34,196
SULT1A4	16p11.2	29,459,913–29,464,966	5054	+	34,196
SULT2A1	19q13.33	47,870,467–47,886,315	15,849	-	33,780
SULT2B1a ^c^	19q13.33	48,552,172–48,599,427	47,256	+	39,599
SULT2B1b ^c^	19q13.33	48,552,172–48,599,427	47,256	+	41,308
SULT4A1	22q13.31	43,824,509–43,862,513	38,005	-	33,085

^a^ According to GRCh38/hg38, NCBI Gene; ^b^ Gene locus size in bases; ^c^ SULT2B1 has two isoforms from same gene are due to different start sites/alternative splicing resulting in different lengths of N-terminal domain, with 2B1b 18 aa longer than 2B1a and 2B1b a major isoform.

**Table 2:** SULT4A1 aa sequence identity conservation.

Figure 4 aa Line *	Vertebrate	% Identical aa
1	Human	100
2	Pongo	99.7
3	Red Fox	99.3
4	Mouse	97.9
5	Sperm Whale	98.9
6	Short-tailed Opossum	95.8
7	Great Tit	94.7
8	Zebrafinch	95.4
9	European Starling	96.1
10	Anole Lizard	96.5
11	Brown Pit Viper	93.0
12	Eastern Brown Snake	93.0
13	High Himalayan Frog	90.8
14	Xenopus	90.1
15	Catfish	87.0
16	Northern Pike	88.3
17	Zebrafish	87.7
18	Whale Shark	87.0

* Sequence alignment shown in Figure 4.

**Table 3:** Human SULT4A1 SNPs ^a^.

SNP	Location
**rs763120**	43826486
rs138060	43826926
rs138067	43831675
rs7291934	43837545
rs138079	43843618
rs470089	43852623
rs2285161	43852720
**rs2285162**	43853582
rs2285164	43854024
rs138097	43854689
**rs2285167**	43855308
rs470091	43855767
rs138099	43855971
rs138102	43857208
rs34601004	43861693
rs138110	43863900

^a^ according to GRCh38/hg38. **Bold**: SULT4A1-1-haplotype-related SNPs.

## Data Availability

No new data were created or analyzed in this study.
